# Soft and energy drinks consumption and associated factors in Saudi adults: a national cross-sectional study

**DOI:** 10.3389/fnut.2023.1286633

**Published:** 2023-12-04

**Authors:** Abeer M. Aljaadi, Abrar Turki, Arwa Z. Gazzaz, Faisal Saeed Al-Qahtani, Nora A. Althumiri, Nasser F. BinDhim

**Affiliations:** ^1^Department of Clinical Nutrition, Faculty of Applied Medical Sciences, Umm Al-Qura University, Mecca, Saudi Arabia; ^2^Clinical Nutrition Department, College of Applied Medical Sciences, University of Hafr Al Batin, Hafar Al Batin, Saudi Arabia; ^3^Department of Periodontics and Community Dentistry, College of Dentistry, King Saud University, Riyadh, Saudi Arabia; ^4^Department of Family and Community Medicine, College of Medicine, King Khalid University, Abha, Saudi Arabia; ^5^Sharik Association for Research and Studies, Riyadh, Saudi Arabia; ^6^Informed Decision Making, Riyadh, Saudi Arabia

**Keywords:** soft drink, energy drink, Saudi Arabia, overweight, adults, consumption, survey, obesity

## Abstract

**Introduction:**

The consumption of soft and energy drinks poses a significant risk for non-communicable diseases, such as diabetes and heart disease. Studies in Saudi Arabia have reported elevated consumption of soft/energy drinks, but nation-wide data is not available. Therefore, this study aims to explore the prevalence of soft and energy drinks consumption and its associated factors among a representative sample of Saudi adults.

**Methods:**

The present research is a secondary data analysis of the 2021 Sharik Diet and Health National Survey (SDHNS). Current analysis used data on socio-demographics, anthropometrics, physical activity, and soft and energy drink consumption. The frequency of soft and energy drinks consumption is assessed on a weekly basis.

**Results:**

Of the 5,194 Saudi adults, 3,928 were analyzed. Overall, 67% consumed soft drinks weekly, while 30% consumed energy drinks weekly. In multiple logistic regression, consumption of either soft drinks or energy drinks was associated with males, a younger age, lower income, and lower physical activity. Individuals with overweight or obesity were less likely to consume energy drinks [OR (95%CI): 0.83 (0.71, 0.99) and 0.73 (0.60, 0.90), respectively] than those with healthy weight. However, education level was not associated with either soft or energy drink consumption. These findings highlight the need for targeted interventions designed to reduce soft and energy drinks consumption in Saudi adults.

## 1 Introduction

Energy drinks, also referred to as power drinks, are liquid refreshments that typically incorporate a variety of ingredients, such as caffeine, sugar, amino acids like taurine, and other energizing substances such as guarana and ginseng (1). These beverages are often promoted because of their apparent advantages, including enhanced energy levels, sharpened mental focus, and improved physical capabilities (2, 3). Nevertheless, they can pose potential health hazards, particularly among youth (4–6).

Conversely, soft drinks are non-intoxicating beverages primarily composed of carbonated water, a sweetening agent, and either natural or synthetic flavorings (7). The sweetener can take various forms, including sugar, high-fructose corn syrup, fruit juice, or artificial sweeteners in the case of diet versions (8–10). Soft drinks can also contain caffeine, colorants, and preservatives, among other constituents. They are a subset of the broader group of sugar-enriched beverages, which have been linked to obesity and other health concerns when ingested in sizable volumes (11, 12).The intake of energy drinks and soft drinks has been progressively associated with adverse health outcomes, including obesity, dental diseases, and type 2 diabetes mellitus (6, 10, 13, 14). Energy drinks have been also associated with arrhythmias, including atrial fibrillation and supraventricular tachycardia, particularly among young consumers due to its high caffeine contents (15–17).

From a behavioristic viewpoint, numerous factors can impact the consumption of energy drinks and soft drinks. These factors encompass taste preferences, the perceived energy surge or mood elevation, influences from marketing and advertising, societal norms, and easy availability (18, 19). The sweet flavor of these beverages, often amplified by their high sugar content, can stimulate the brain’s reward system, promoting habitual consumption (20, 21). Energy drinks are frequently consumed due to their supposed benefits in boosting energy and alertness, appealing to individuals coping with intense work or academic stress, or those aiming to augment physical performance (22). Advertisements and marketing campaigns often depict these beverages as stylish, enjoyable, or vital for peak performance, thereby influencing consumer attitudes and actions. Moreover, the ubiquitous presence and societal acceptance of these drinks in different contexts (like social gatherings, workplaces, and vending machines) can encourage regular consumption (23).

The strategy of increasing taxes on energy drinks and soft drinks is a policy that has been implemented by Saudi Arabia in 2017 to curb overconsumption and alleviate related health implications (24, 25). This tactic is based on the principle that pricing significantly influences consumer habits (26). Preliminary studies indicate that such taxation increased prices and reduced purchasing, but whether this reduced consumption in Saudi Arabia remains unclear (25).

A number of factors contribute to the consumption of soft and energy drinks worldwide. For example, consumption of soft or energy drinks is commonly associated with males, young people, and people of lower socioeconomic status (26, 27). Moreover, individuals with higher BMI or physically inactive people are more likely to consume soft and energy drinks (28). Understanding these factors facilitates targeted interventions to reduce consumption and improve public health. Nonetheless, to date, national prevalence data regarding the consumption of soft and energy drinks among Saudis and their associated factors are lacking. We aim in this study to determine the consumption of soft and energy drinks among Saudi adults and investigate associated factors.

## 2 Materials and methods

### 2.1 Study design and sampling

This study is a secondary data analysis of the 2021 Sharik Diet and Health National Survey (SDHNS) (29). The SDHNS, an annual nationwide cross-sectional survey conducted in Saudi Arabia, employs phone interviews (29). For a balanced distribution of participants, the SDHNS uses a proportional quota sampling method, stratified by age, gender, and region across Saudi Arabia’s 13 administrative regions. The SDHNS integrates ZDataCloud^®^, a data collection tool that minimizes sampling bias without human interaction (30). The dataset used in this research was collected in 2021 encompassing *n* = 5,194 participants. A detailed methodology of the SDHNS is available in a separate document published by the Sharik Association for Research and Studies (29). Ethical approval was obtained from the ethics committee of Sharik Association for Research and Studies (Approval no.06–2021).

### 2.2 Measurements

The weekly consumption was measured based on the frequency of the consumption of soft and energy drinks in separate questions. Participants were asked “During the past week, how many times per week have you consumed soft drinks?” and “During the past week, how many times per week have you consumed energy drinks?.” Answers ranged from “none” to “seven times per week.” Participant answers were classified into two groups for the regression analyses: those who drunk any soft drink per week (“none” = 0) and at least once per week (“yes” = 1). Similar classification was used for energy drinks consumption question.

Sociodemographic information, including age, sex, education level, and monthly income, was provided by the SDHNS database. Level of income was measured by asking the participant about the monthly range of their income. The possible answers were: (1) I do not have stable monthly income, (2) less than SR 5000, (3) between SR 5,000 and SR 16,000, (4) more than SR 16,000. Those who answered “I do not have stable income” were excluded, *n* = 1,239 as we were interested in gradient relationship across income categories. Education was measured by asking the participant about the highest attainable degree.

BMI was calculated from self-reported height and weight. Weight status was classified as underweight (BMI <18.5 kg/m^2^), healthy (BMI: 18.5–24.9 kg/m^2^), overweight (25–29.9 kg/m^2^), and obesity (BMI ≥ 30 kg/ m^2^) (31). Extreme BMI values were excluded (<15 kg/m^2^ or ≥ 50 kg/ m^2^). The participants’ level of physical activity adhered to the World Health Organization’s (WHO) recommendations, which include engaging in moderate-intensity activity for at least 30 or 20 min of high-intensity activity in the past week (32). Subsequently, the activity level was categorized as: (1) none: when the participants did not engage in any type of physical activity, (2) less than recommended level: when the participants engage in some physical activity but did reach the recommended level and (3) follow recommended level: when the participants met the WHO recommendations (32).

### 2.3 Statistical analyses

Categorical variables are presented as frequencies (n) and percentages (%) across four levels of weekly consumption: never, 1–2 times, 3–4 times, and 5 times or more per week. Bivariate analyses were conducted using a chi- square test of association (proportions) to identify factors associated with weekly consumers versus non-consumers. Logistic regression models were used to identify factors and the magnitude of association with frequency of soft or energy drinks consumption. The outcome was categorized as the consumption of at least one time per week or none. Factors that showed significant association in the bivariate analyses with weekly soft or energy consumption (*p* < 0.05) were included in the adjusted models.

## 3 Results

The study sample included *n* = 5,194 participants. Participants who reported unstable income (*n* = 1,239, 24%) or extreme BMI values (*n* = 45, <1%) were excluded. Overall, a total of *n* = 3,928 were included in the analyses, 57% of which were males, 43% were 30–49 years, 65% had a post-secondary education, and 54% had overweight or obesity (BMI ≥ 25 kg/m^2^).

### 3.1 Soft drinks consumption

Soft drinks consumption is shown by participants’ characteristics in Table 1. About 67% reported consuming soft drinks at least once a week, with more males than females reporting weekly consumption (69% vs. 65%, *p* < 0.01). Consumption of soft drinks also differed by age, monthly income, physical activity level, and weight status. Education level did not differ between consumers and non-consumers.

**Table 1:** Characteristics of study sample by soft drinks consumption.

	Soft drinks consumption				*P*-value
	Never	1–2 times/week	3–4 times/week	≥5 times/week	
Total	1,288 (32.3)	1,150 (29.3)	759 (19.3)	731 (18.6)	
Gender					
Male	694 (31.1)	597 (26.8)	451 (20.2)	490 (22.0)	0.009
Female	594 (35.0)	553 (32.6)	308 (18.2)	241 (14.2)	
Age group					
<30 years	309 (23.0)	361 (26.9)	304 (22.6)	370 (27.5)	0.000
30–49 years	503 (29.8)	537 (31.8)	357 (21.12)	293 (17.3)	
≥50 years	476 (53.2)	252 (28.19)	98 (11.0)	68 (7.6)	
Education level					
≤High school	467 (34.1)	374 (27.3)	272 (19.9)	256 (18.7)	0.154
Diploma and bachelor	748 (31.6)	719 (30.4)	452 (19.1)	449 (19.0)	
Master and PhD	73 (38.2)	57 (29.84)	35 (18.3)	26 (13.6)	
Income level					
<5,000 SAR	436 (29.9)	419 (28.7)	293 (20.1)	311 (21.3)	0.000
5,000–16,000 SAR	621 (31.5)	604 (30.7)	379 (19.2)	366 (18.6)	
>16,000 SAR	231 (46.3)	127 (25.5)	87 (17.4)	54 (10.8)	
Physical activity*					
None	419 (32.7)	370 (28.8)	226 (17.6)	268 (20.9)	0.000
Less than recommended level	512 (28.8)	584 (32.8)	384 (21.6)	299 (16.8)	
Follow recommended level	357 (41.2)	196 (22.6)	149 (17.2)	164 (18.9)	
BMI^‡^					
Underweight	44 (24.4)	62 (34.4)	32 (17.8)	42 (23.3)	0.001
Healthy weight	506 (31.0)	445 (27.3)	342 (21.0)	337 (30.7)	
Overweight	463 (35.1)	399 (30.3)	258 (18.8)	208 (15.8)	
Obesity	275 (34.4)	244 (30.5)	137 (17.1)	144 (18.0)	

Data are presented as n (%). Proportions are row total. *Includes intense and moderate. ^‡^BMI was used to define weight status as follows: underweight (BMI <18.5 kg/m^2^), healthy (BMI: 18.5–24.9 kg/m^2^), overweight (25–29.9 kg/m^2^), and obesity (BMI≥30 kg/m^2^).

### 3.2 Energy drinks consumption

Energy drinks consumption is shown by participants characteristics in Table 2. Nearly, 30% reported consuming energy drinks at least once a week, with more males than females reporting weekly consumption (34% vs. 25%, *p* < 0.001). Significant differences in consumption of energy drinks were observed by age, monthly income, physical activity level, and weight status. Education level did not differ between consumers and non-consumers.

**Table 2:** Characteristics of study sample by energy drinks consumption.

	Energy drinks Consumption				
	Never	1-2 times/week	3-4 times/week	≥ 5 times/week	*P* value
Total	2,747 (69.9)	667 (17.0)	343 (8.7)	171 (4.4)	
Gender					
Male	1,466 (65.7)	431 (19.3)	219 (9.8)	116 (5.2)	0.000
Female	1,281 (75.5)	236 (13.9)	124 (7.3)	55 (3.2)	
Age group					
<30 years	813 (60.5)	307 (22.8)	143 (10.6)	81 (6.03)	0.000
30–49 years	1,186 (70.2)	284 (16.8)	150 (8.9)	70 (4.1)	
≥50 years	748 (83.7)	76 (8.5)	50 (5.6)	20 (2.2)	
Education level					
≤High school	960 (70.1)	221 (16.1)	135 (9.9)	53 (3.9)	0.086
Diploma and bachelor	1,652 (69.8)	421 (17.8)	185 (7.8)	110 (4.7)	
Master and PhD	135 (70.7)	25 (13.1)	23 (12.0)	8 (4.2)	
Income level					
<5,000 SAR	985 (67.5)	289 (29.8)	116 (8.0)	69 (4.7)	0.000
5,000–16,000 SAR	1,353 (68.9)	331 (16.8)	197 (10.0)	89 (4.5)	
>16,000 SAR	409 (82.0)	47 (9.4)	30 (6.0)	13 (2.6)	
Physical activity*					
None	926 (72.2)	206 (16.1)	91 (7.1)	60 (4.7)	0.000
Less than recommended level	1,168 (65.7)	365 (20.5)	183 (10.3)	63 (3.5)	
Follow recommended level	653 (75.4)	96 (11.1)	69 (8.0)	48 (5.5)	
BMI‡					
Underweight	112 (62.2)	40 (22.2)	17 (9.4)	11 (6.1)	0.000
Healthy weight	1,068 (65.5)	315 (19.3)	158 (9.7)	89 (5.5)	
Overweight	953 (72.3)	192 (14.6)	117 (8.9)	56 (4.3)	
Obesity	614 (76.8)	120 (15.0)	51 (6.4)	15 (1.9)	

Data are presented as n (%). Proportions are row total. *Includes intense and moderate. ^‡^BMI was used to define weight status as follows: underweight (BMI < 18.5 kg/m^2^), healthy (BMI: 18.5–24.9 kg/m^2^), overweight (25–29.9 kg/m^2^), and obesity (BMI ≥ 30 kg/m^2^).

### 3.3 Factors associated with soft and energy drinks consumption

Table 3 presents unadjusted and adjusted logistic regression models of factors associated with weekly soft drinks consumption. Females had lower odds of consuming soft drinks weekly compared to males [crude OR (95%CI) = 0.84 (0.73, 0.96)]. Adults over the age of 50 y and those 30–49 y were less likely to consume soft drinks [crude ORs (95%CI): 0.26 (0.22, 0.31) and 0.70 (0.60, 0.83), respectively] compared to younger adults <29 y. Overweight, but not obesity, was associated with lower odds of soft drinks consumption compared to healthy weight adults [crude OR (95%CI): 0.83 (0.71, 0.97)]. Participants with higher income >16,000 SAR and those following physical activity recommendations had lower odds of consuming soft drinks consumption than reference categories (<5,000 SAR and no physical activity, respectively). Interestingly, those who performed physical activity less than the recommended level had higher odds of consuming soft drinks than those with no physical activity. In the fully adjusted model, gender, age, monthly income, and physical activity remained significantly associated with soft drinks consumption following the same direction of association. Weight status was not associated with soft drinks consumption after adjusting for age, gender, monthly income, and physical activity.

**Table 3:** Odd ratios of factors associated with soft drink consumption and their 95% confidence intervals (*n* = 3,928).

	Weekly soft drinks consumption	
	Model 1	Model 2
	OR (95% CI)	AOR (95% CI)
Gender		
Male	1.00	1.00
Female	0.84 (0.73, 0.96)**	0.69 (0.60, 0.80)***
Age group		
<30 years	1.00	1.00
30–49 years	0.70 (0.60, 0.83)***	0.65 (0.54, 0.79)***
≥50 years	0.26 (0.22, 0.31)***	0.24 (0.19, 0.29)***
Education level		
≤High school	1.00	–
Diploma and bachelor	1.12 (0.96, 1.29)	–
Master and PhD	0.84 (0.61, 1.14)	–
Income level		
<5,000 SAR	1.00	1.00
5,000–16,000 SAR	0.93 (0.80, 1.07)	1.13 (0.95, 1.34)
>16,000 SAR	0.49 (0.40, 0.61) ***	0.72 (0.57, 0.91) **
Physical activity		
None	1.00	1.00
Less than recommended level	1.20 (1.03, 1.40)*	1.18 (1.00, 1.38)*
Follow recommended level	0.69 (0.58, 0.83)***	0.60 (0.50, 0.73)***
BMI^‡^		
Healthy weight	1.00	1.00
Underweight	1.39 (0.97, 1.99)	1.13 (0.78, 1.64)
Overweight	0.83 (0.71, 0.97)*	0.99 (0.84, 1.17)
Obesity	0.86 (0.72, 1.03)	1.16 (0.95, 1.40)

*p<0.05; **p<0.01; ***p<0.001. Results obtained from binary logistic regression models. Model 1 column reports crude odds ratio; Model 2 columns report estimate from models mutually adjust for gender, age group, income level, physical activity and BMI. AOR, adjusted odds ratio; CI, confidence interval.

Table 4 illustrates logistic regression analyses of unadjusted and adjusted odds ratios of factors associated with weekly energy drinks consumption. Similar to the pattern observed in soft drinks consumption, females had lower odds of consuming energy drinks weekly compared to males [AOR (95%CI): 0.53 (0.46, 0.62)]. Adults over the age of 50 y and 30–49 y were less likely to consume soft drinks compared to younger adults <29 y. Both overweight and obesity were associated with lower odds of energy drinks consumption compared to adults with healthy weight. Higher income >16,000 SAR was associated with lower odds of consuming energy drinks than those who earned <5,000 SAR. Education level was not associated with weekly energy drinks consumption as observed in soft drinks. Following physical activity recommendations was associated with lower odds of consuming energy drinks; however, less than the recommended physical activity was associated with higher odds of consumption than the reference category (no physical activity). In the fully adjusted model, gender, age, monthly income, weight status, and physical activity remained significantly associated with energy drinks consumption following the same direction of association in the bivariate analyses.

**Table 4:** Odd ratios of factors associated with energy drink consumption and their 95% confidence intervals (*n* = 3,928).

	Weekly energy drinks consumption	
	Model 1	Model 2
	OR (95% CI)	AOR (95% CI)
Gender		
Male	1.00	1.00
Female	0.62 (0.54, 0.71)***	0.53 (0.46, 0.62)***
Age group		
<30 years	1.00	1.00
30–49 years	0.65 (0.56, 0.76)***	0.65 (0.55, 0.78)***
≥50 years	0.30 (0.24, 0.37)***	0.31 (0.24, 0.39)***
Education level		
≤High school	1.00	–
Diploma and Bachelor	1.02 (0.88, 1.18)	–
Master and PhD	0.97 (0.70, 1.36)	–
Income level		
<5,000 SAR	1.00	1.00
5,000–16,000 SAR	0.95 (0.82, 1.10)	1.08 (0.91, 1.28)
>16,000 SAR	0.46 (0.36, 0.59)***	0.60 (0.45, 0.79)***
Physical activity		
None	1.00	1.00
Less than recommended level	1.36 (1.16, 1.59)***	1.33 (1.13, 1.56)***
Follow recommended level	0.85 (0.69, 1.03)	0.72 (0.59, 0.89)**
BMI^‡^		
Healthy weight	1.00	1.00
Underweight	1.15 (0.84, 1.59)	1.02 (0.74, 1.42)
Overweight	0.73 (0.62, 0.85)***	0.83 (0.71, 0.99)*
Obesity	0.58 (0.47, 0.70)***	0.73 (0.60, 0.90)**

*p < 0.05; **p < 0.01; ***p < 0.001. Results obtained from binary logistic regression models. Model 1 column reports crude odds ratio; Model 2 columns report estimate from models mutually adjust for gender, age group, income level, physical activity and BMI. AOR, adjusted odds ratio; CI, confidence interval.

## 4 Discussion

Based on nationally representative data of Saudi adults, weekly consumption of soft drinks was 67% and energy drinks consumption was 30%. Analyses showed that Saudi females were less likely to consume soft and energy drinks compared to males and that adults less than 30 years old had the highest prevalence of consuming both soft and energy drinks. Interestingly, adherence to the WHO’s physical activity recommendations was inversely correlated with the consumption of soft and energy drinks. Similarly, adults with obesity exhibited a lower likelihood of weekly consumption of energy drinks. However, weight status was not associated with soft drinks consumption after adjusting for age, gender, income, and physical activity level.

In this study, the prevalence of soft drinks consumption in Saudi adult population was 67% in any given week. Our findings support the evidence that Saudi Arabia is one of the biggest consumers of soft drinks in the Middle East region (33). Globally, Saudi Arabia ranks fifth in terms of calorie consumption derived from sugar-sweetened beverages (33). The findings of the current study are consistent with findings from a national study showing that ~71% of Saudi adults consume sugar-sweetened beverages, which include soft drinks, at least once a week using data collected in 2013, with 36% of adults consume these beverages on a daily basis (34). Saudi Arabia implemented a 50% tax on sugary drinks, including soft drinks in 2017, which aligns with a broader initiative to combat the escalating rates of obesity and type 2 diabetes mellitus, conditions correlated with the overconsumption of these beverages (24, 35). A 2019 study on 1,194 adults living in Saudi Arabia reported a lower rate of weekly soft drinks consumption (44%); of which, 6% reported consuming soft drinks daily (36). Consumption of soft drinks on a daily basis has been observed in developed nations, such as the UK (20.4%) (37) and the USA (40.0%) (38), with similar prevalence in South Africa (48.3%) (39). This may be due to the preference for fast food and soft drinks consumption in western and European countries. A trend toward a busy lifestyle with less emphasis on finding time to cook may also be emerging (40).

We found that around 30.0% Saudi adults consume energy drinks on a weekly basis, which is in agreement with the most recent study performed among Saudi young adults (29.3%) (41). However, the results of the present study are inconsistent and lower compared with earlier studies executed in Saudi Arabia. A 2017 study that included students from the Prince Sattam bin Abdulaziz University found that 81.3% of students consumed energy drinks, while in another study conducted at the University of Dammam, 45.3% of participants consumed energy drinks (42, 43). Both studies, however, did not report a clear frequency of consumption to facilitate comparison. Compared to Malaysian adults, weekly consumption appears to be higher in our study (30.0% vs. 18.8%) (27). It is suggested that these reduced consumption in recent years is due to the new rules of the Saudi Food and Drug Authority (SFDA) and the General Authority of Zakat and Tax (GAZT) related to energy drinks. In 2017, SFDA published few rules for handling energy drinks; one of these rules includes that manufacturers must write the warning phrases on the package (44). Weekly consumption has been observed in the 16 European countries (16.0%) (45) and Denmark (15.8%) (26). Energy drinks are considered new in the Danish soft drink market, which may be one reason why young adults in Denmark are less likely to consume energy drinks. Additionally, the above drinks tend to be more expensive than other drinks, which may also contribute to this phenomenon (26).

In the current study, gender, age, monthly income, and physical activity were identified as contributing factors to soft and energy drinks consumption. Gender was found to be significantly associated with soft and energy drinks consumption. Females had lower odds for consuming soft and energy drinks weekly [AOR (95%CI): 0.69 (0.60, 0.80) vs. AOR (95%CI): 0.53 (0.46, 0.62), respectively] compared to males. This finding is consistent with findings from several earlier population-based studies in Saudi Arabia (34), USA (38), and Australia (46), which revealed that males were more likely to consume soft and energy drinks than females. A study in Denmark found 25% of men consume energy drinks on a weekly basis, suggesting that the consumption of energy drink is predominantly observed among males (26). Another study in Cambodia found the men are more likely [AOR (95%CI): 1.49 (1.10, 2.00)] to consume soft drinks than women (40) This difference could be attributed to gender-driven roles, identities, and social norms. For example, men may be more likely to be influenced by advertising that targets males. It could be also that females tend to more health-conscious and the desire to be a positive role-model. Previous research in several populations (34, 41, 47, 48) has demonstrated a strong correlation between the age of an individual and their consumption of soft and energy drinks, and the current study’s findings support this. Our result was that young adults <29 y are more likely to consume soft and energy drinks compared to adults over the age of 50 y and those 30–49 y. However, a study on Cambodian adults opposes this finding (40).

Monthly income was significantly associated with soft and energy drinks consumption. Those who earned higher income >16,000 SAR per month were less likely to consume soft and energy drinks compared to those who earned <5,000 SAR per month. This observation is in line with data from Singapore, which indicates that high and middle-income individuals consume fewer soft drinks in comparison to lower-income individuals (48). In addition, a 2022 Saudi study indicated that adults with higher incomes were less likely to consume soft drinks (34). Conversely, a study conducted in Australia found that individuals with a higher annual income tended to consume more soft drinks than those with a lower annual income (39). The reason that higher income participants in this study were less likely to consume soft and energy drinks could be explained by how high income can increase people access to knowledge, material, cultural, and psychosocial resources that can influence their engagement in such behaviors (49). However, our study did not observe any association between education level and energy and soft drinks consumption. Existing evidence indicates that the consumption of energy drinks was more common among individuals with lower educational attainment levels than those with higher educational attainment levels (26). Thus, examining several socioeconomic status indicators, such as income and education, can improve our understanding of how such indicators influence soft and energy drinks consumption, as they usually represent different social processes in health and health behaviors (50).

As expected, adhering to physical activity recommendations was associated with lower odds of consuming soft and energy drinks. This conclusion is in line with the findings of a population-based cross-sectional survey conducted in Spain, which showed that the prevalence of diet soda consumption was highest among inactive adolescents (51). Adolescents may consume diet soda in response to the realization that they are not consuming enough energy (51). In the context of our study in Saudi adults, it might be that participants who are following physical activity recommendations consuming less soft and energy drinks in order to follow healthy food choices and maintain overall health. Regarding weight status, we found that overweight/obesity were associated with lower odds of energy drinks consumption compared to adults with healthy weight. This result is inconsistent with previous evidence, which might reflect differences in population and methodology (52, 53). In three Eastern European cohorts have indicated positive correlation between consumption of sugar-sweetened beverage and BMI (52). A study on 515 Malaysian adults reported no bivariate association between energy drinks consumption and BMI (27). A recent systematic review of 26 observational studies showed that only one study reported no correlation between the intake of sugar-sweetened beverage and weight gain (53). It is possible that participants classified as overweight/obese in our study were avoiding the consumption of energy drinks for better weight control or under-reported consumption, causing social desirability bias and this needs to be further explored.

Advertisements and marketing campaigns frequently portray energy drinks as fashionable, pleasurable, or necessary for optimal performance, affecting consumer behaviors and attitudes (23, 54). A study in Canada indicated a considerable number of energy drink postings on social media in 2020–2021, with several posts have using marketing strategies on Twitter, Facebook and Instagram, that may be appealing to adolescents. Another study in Saudi Arabia found that advertising is the primary source of information regarding energy drinks among 43% of Saudi adolescents (55). However, a recent study on n = 316 students from two Saudi universities reported no associations between social media platforms or watching relevant social media advertisements with sugary drinks consumption after adjusting for age, sex, nationality, marital status, academic year, monthly household income, and BMI; weekly users of Snapchat had lower odd of consuming sugary drinks than daily user 0.33 (0.11–0.98) (56). Further studies are needed to understand the impact of the media on soft and energy drinks consumptions in the Saudi Arabian context.

Finally, the present study measures the consumption of soft and energy drinks by asking the participants about their frequency of intake each week rather than the actual quantity of consumption. Using the frequency of consumption, as an index of actual amount consumption is easier and a relatively reliable tool to judge the amount of consumption which was adopted in many other studies (57–59). Studies have shown correlations between frequency of consumption and quantity of soft drinks consumption (57–59). Frequency of consumption has been associated with adverse health behaviors and higher rates of overweight/obesity among children, adolescents, and adults from several countries (6, 60, 61).

The current study has many strengths, which includes a relatively large sample size representative of Saudi adults from all regions of Saudi Arabia by using multistage quota sampling. This sampling technique was limiting the risk of selection bias and allowing for the recruitment of a balanced study sample in terms of gender and age that representing all the regions of the kingdom. On the other hand, the use of a research participant database might introduce bias, given that participation in the database was voluntary. Data integrity checks, inherent to the QPlatform data collection system, minimize invalid or erroneous data entry. The study was also able to report on soft drinks and energy drinks separately. However, the study has some limitations. The cross-sectional design might affect the causative relationship between the soft and energy drinks consumption and possible associations. The self-reported data is commonly used in nutrition research and public health surveillance, but it is vulnerable to recall bias and social desirability bias, which may result in misreporting particularly among those who are overweight. Furthermore, the soft and energy drinks consumption was measured by frequency rather than actual quantity that ingested by participants on the weekly basis. Data was not available on the sizes or types of soft and energy drinks; some might be consuming non-sugar sweetened drinks that have a different impact compared to sugar-sweetened beverages.

## 5 Conclusion

Overall, this study on a representative sample of Saudi adults shows that 67% consume soft drinks and 30% consume energy drinks weekly. Males, younger adults, and those with higher incomes are more likely to consume soft drinks weekly than females, older adults, and those with lower incomes. The soft drinks consumption remains high despite the implementation of taxes and alarm action, particularly among young adults and men. Studies are needed on the type and quantity of drinks consumed and in-depth analyses of associated factors; this could inform targeted interventions for any future programs on the prevention and/or management of non-communicable diseases. The findings of this study highlight the need for public health interventions to the consumption of soft drinks and energy drinks in Saudi Arabia. These interventions should be based on a comprehensive understanding of the factors that influence consumption and tailored to the specific needs of the Saudi population.

## Data availability statement

The raw data supporting the conclusions of this article will be made available by the authors, without undue reservation.

## Ethics statement

The studies involving humans were approved by Ethics Committee of Sharik Association for Research and Studies (Approval no. 06–2021). The studies were conducted in accordance with the local legislation and institutional requirements. The participants provided their written informed consent to participate in this study.

## Author contributions

AA: Conceptualization, Investigation, Supervision, Writing – original draft, Writing – review & editing. AT: Investigation, Writing – original draft, Writing – review & editing. AG: Formal analysis, Investigation, Software, Writing – review & editing. FA-Q: Conceptualization, Writing – original draft, Writing – review & editing. NA: Methodology, Resources, Writing – original draft, Writing – review & editing. NB: Data curation, Funding acquisition, Resources, Writing – review & editing.
